# Common mistakes in reporting systematic reviews and meta-analyses

**DOI:** 10.34172/hpp.2020.17

**Published:** 2020-03-30

**Authors:** MohammadBagher Shamsi, Maryam Mirzaei, Siavash Vaziri, Hamid Reza Mozaffari

**Affiliations:** ^1^School of Allied Medical Sciences, Kermanshah University of Medical Sciences, Kermanshah, Iran; ^2^Department of Infectious Diseases, Kermanshah University of Medical Sciences, Kermanshah, Iran; ^3^Department of Oral and Maxillofacial Medicine, Kermanshah University of Medical Sciences, Kermanshah, Iran

We have read the interesting article by Moradi et al. “A systematic review and meta-analysis on incidence of prostate cancer in Iran” carefully.^1^ It seems that this review, as many other health research reviews published, do not pay proper attention to the reporting quality of systematic reviews according to the Preferred Reporting Items for Systematic Reviews and Meta-Analyses (PRISMA) guideline.

1. Google Scholar was considered as one of the databases in the literature search section. It should be defined as a “search engine”. It is important for the reviewers to specify that which one is a database and which one is a search engine.
2. In the text or in the aims of the study the authors followed the incidence of prostate cancer, while in search strategy of the study they addressed the “prevalence” and “incidence” keywords. The main aim of the study was to investigate the “incidence”, so the “prevalence” should not be included in the keywords.
3. The authors illustrated that they included 9 evidences. The ﬂowchart of the study selection, includes some mistakes. After excluding 12 unrelated evidences, they indicated that 9 evidences remained in the qualitative/ quantitative synthesis; however, 7 evidences should remain. The numbers of the next steps are incorrect.
4. Another point is about importing the quality assessment of included evidences (risk of bias within studies). The methodological quality assessment is a key concept; Based on PRISMA statement, it is recommended to include the results of assessing the methodological quality of each evidence; but in this present form, the interpretation of results was presented without making any attempt about the quality assessment.^2,3^
5. The authors stated that used Begg’s test, Egger’s test and funnel plot for assessing the publication bias. Based on evidence there is no need for conducting these tests for systematic review of incidence/prevalence studies. It is important to recognize that these tests describe the potential influence of publication bias on the calculated effect size, in clinical trial studies.^2,4^

In this letter, we discussed some common mistakes in reporting systematic reviews and meta-analyses. Author’s adherence to reporting guidelines is an important point in improving the reporting quality of systematic reviews.^2^

## Ethical approval

Not applicable.

## Competing interests

The authors declare that they have no competing interests.

## Authors’ contributions

MBSh and MM contributed in original idea, conception of the work, wrote and editing of this manuscript. SV and HRM contributed in the conception of the work, and editing of this manuscript. All authors read and approved the final manuscript.
